# Eplerenone vs. spironolactone in heart failure: long-term cardiorenal and mortality outcomes in a registry-based cohort

**DOI:** 10.1093/ehjcvp/pvag064

**Published:** 2026-09-10

**Authors:** Karl Wärnberg, Carl Haggård, Karin Bergen, Henrik Falhammar, Buster Mannheimer, Krister Lindmark

**Affiliations:** Department of Clinical Science, Danderyd Hospital, Karolinska Institutet, 182 88 Stockholm, Sweden; Department of Cardiology, Danderyd Hospital, 182 88 Stockholm, Sweden; Department of Clinical Science, Danderyd Hospital, Karolinska Institutet, 182 88 Stockholm, Sweden; Department of Cardiology, Danderyd Hospital, 182 88 Stockholm, Sweden; Department of Clinical Science, Danderyd Hospital, Karolinska Institutet, 182 88 Stockholm, Sweden; Department of Nephrology, Danderyd Hospital, 182 88 Stockholm, Sweden; Department of Molecular Medicine and Surgery, Karolinska Institutet, 171 76 Stockholm, Sweden; Department of Endocrinology, Karolinska University Hospital, Stockholm, 171 76 Sweden; Department of Clinical Science and Education at Södersjukhuset, Karolinska Institutet, 118 83 Stockholm, Sweden; Department of Medicine, Section of Diabetes and Endocrinology, Södersjukhuset, 118 83 Stockholm, Sweden; Department of Clinical Science, Danderyd Hospital, Karolinska Institutet, 182 88 Stockholm, Sweden; Department of Cardiology, Danderyd Hospital, 182 88 Stockholm, Sweden

**Keywords:** Mineralocorticoid receptor antagonist, Mortality, Hospitalization, Kidney, Hyperkalaemia, Congestive cardiac insufficiency

## Abstract

**Aims:**

Eplerenone and spironolactone differ in pharmacological profile, but whether this translates into meaningful differences in clinical outcomes in heart failure (HF) remains uncertain. We compared the two agents in a registry-based new-user cohort with serial laboratory data.

**Methods and results:**

In this observational cohort study, adults with a first HF diagnosis in Stockholm County (2005–2019) who were prescribed either spironolactone or eplerenone within 6 months were identified from linked Swedish registries with individual-level laboratory results. Follow-up was analysed under an intention-to-treat principle. Outcomes included all-cause mortality, HF hospitalization, kidney function decline, and hyperkalaemia. The main analysis used multivariable Cox regression. Several sensitivity analyses were performed, including propensity score matching. Of 75 565 patients with new-onset HF, 12 046 initiated a mineralocorticoid receptor antagonist (11 039 spironolactone; 1007 eplerenone). Eplerenone was associated with lower all-cause mortality (hazard ratio 0.78, 95% confidence interval 0.62–0.98), a lower risk of more than 50% decline in estimated glomerular filtration rate (eGFR) (0.69, 0.54–0.88), sustained eGFR below 30 mL/min/1.73 m^2^ (0.69, 0.49–0.95), a composite kidney outcome (0.71, 0.56–0.90), and hyperkalaemia of 5.5 mmol/L or above (0.76, 0.60–0.96). The cause-specific hazard for HF hospitalization was higher with eplerenone (1.29, 1.11–1.51) but attenuated to 0.99 (0.87–1.12) when death was treated as a competing risk. Findings were robust across sensitivity analyses.

**Conclusion:**

Compared with spironolactone, eplerenone was associated with lower all-cause mortality and lower risks of kidney function decline and mild hyperkalaemia. These observational findings warrant confirmation in randomized comparisons.

## Graphical Abstract

**Graphical Abstract:**
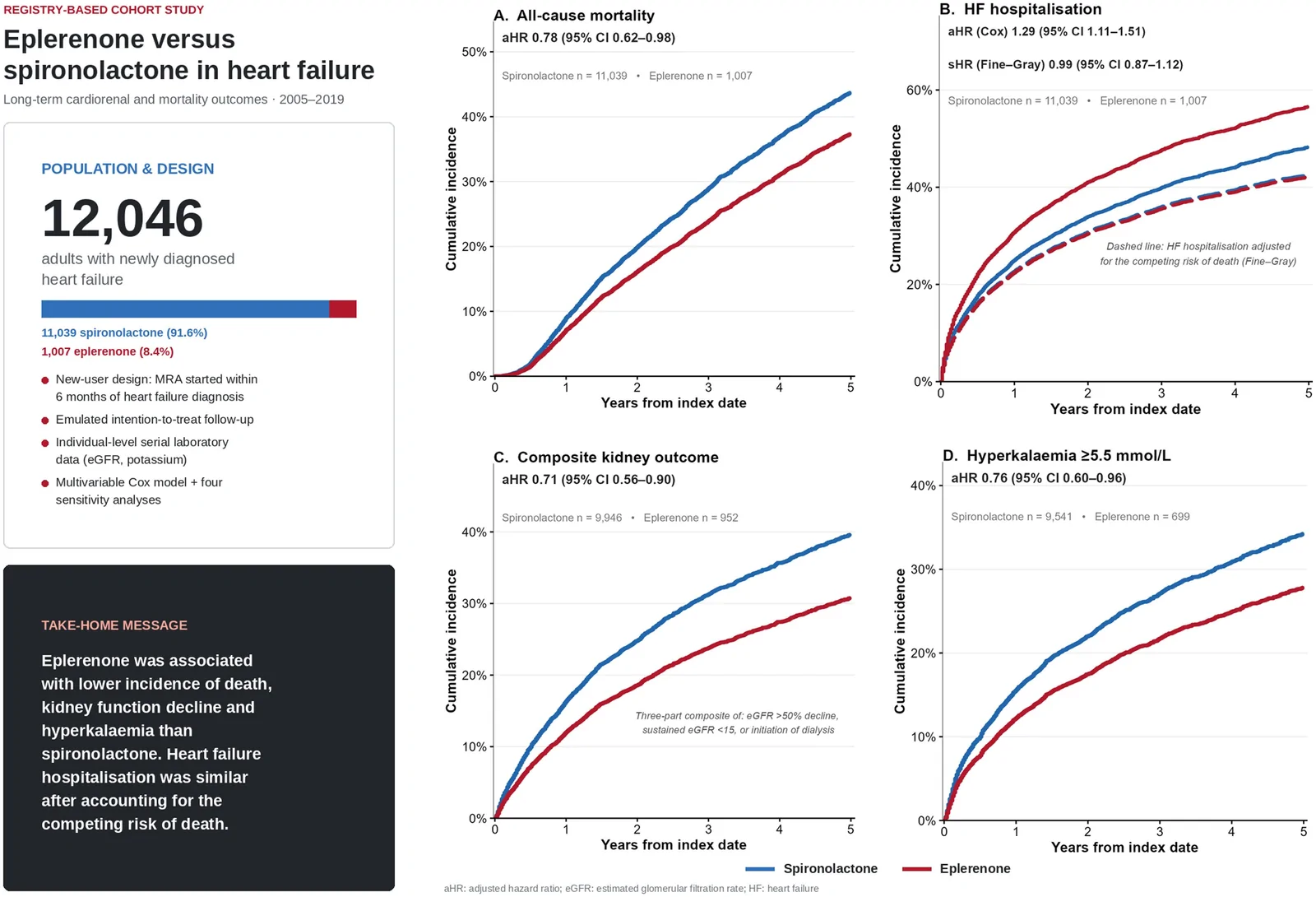

## Introduction

Mineralocorticoid receptor antagonists (MRAs) are a cornerstone of heart failure (HF) therapy. In landmark randomized placebo-controlled trials, spironolactone and eplerenone both reduced morbidity and mortality compared with placebo, and more recent evidence has extended MRA benefit across the ejection fraction spectrum.^1–5^ In current guidelines, MRAs carry a Class I recommendation for HF with reduced ejection fraction and are increasingly used more broadly across the entire ejection fraction spectrum.^6,7^

Pharmacologically, spironolactone and eplerenone share the same mechanism of action but differ in selectivity. Eplerenone acts selectively on the mineralocorticoid receptor, whereas spironolactone also binds androgen and progesterone receptors. Spironolactone also has a longer half-life and more active metabolites.^8^ It remains uncertain whether these pharmacological differences translate into clinically meaningful differences in cardiovascular (CV) and kidney outcomes.^9^

In the last 5–10 years, a growing body of evidence has begun to address this question. Small randomized trials have suggested more favourable effects of eplerenone on echocardiographic parameters of left ventricular function,^10,11^ supported by a recent meta-analysis of cardiac remodelling endpoints^12^ and by observational data from a smaller single-centre cohort.^13^ A nationwide Danish cohort reported a directionally similar but non-significant difference in mortality,^14^ and a subsequent Danish target-trial emulation in patients with echocardiographically confirmed HF with reduced ejection fraction found significantly lower mortality with eplerenone.^15^ This emerging evidence, however, has been confined to patients with reduced ejection fraction, follow-up has rarely exceeded 1–2 years, and no study has incorporated individual-level laboratory data on kidney function or potassium. The comparative long-term safety of the two agents, in particular their effects on kidney function and hyperkalaemia in routine care, remains undefined.

We conducted a cohort study using linked Swedish registry data with comprehensive individual-level laboratory records, to compare the long-term effectiveness and safety of eplerenone vs. spironolactone. Particular attention was given to kidney function and hyperkalaemia over extended follow-up.

## Methods

### Data source

We used data from the Stockholm Sodium Cohort (SSC), comprising all residents of Stockholm County, Sweden (population around 2 million), who had undergone plasma or serum sodium testing at any point between 1 January 2005 and 31 December 2019.^16^ Sodium testing is very common in routine care, and the SSC therefore covers ∼64% of the adult population of Stockholm County, and 97% of individuals aged 80 years or older. These coverage figures refer to the SSC source population. The analysed cohort described below is a subset of SSC participants with incident HF who initiated an MRA.

The SSC is linked at the individual level to five data sources: regional clinical laboratories, the Swedish National Patient Register, the Swedish Prescribed Drug Register, the Swedish Cause of Death Register, and national demographic registers. Follow-up was censored on 31 December 2019.

### Study design and population

This was an observational, retrospective cohort study with an active-comparator, new-user design. The choice between spironolactone and eplerenone was made by the treating clinician during routine care and was ascertained retrospectively from dispensing records. We identified adults aged 18 years or older with a first HF diagnosis between 2005 and 2019. Heart failure was defined by ICD-10 codes I50, I11.0, I13.0, I13.2, or I42.6. Only patients with a primary HF diagnosis were included; secondary HF diagnoses were not included.

A 6-month enrolment window opened on the date of the first HF diagnosis. Each patient was allocated to an exposure group according to which MRA their treating clinician prescribed at the index date, and retained that assignment throughout follow-up. The index date was the date of first MRA dispensing. Follow-up was analysed under an emulated intention-to-treat principle. Subsequent dose changes, switching, and discontinuation did not alter group assignment.

Patients were excluded under three criteria. First, any MRA dispensing in the 12 months before the HF diagnosis was an exclusion. This preserved a new-user design. Second, death within the 6-month enrolment window was an exclusion criterion to avoid immortal time bias. Third, patients with zero or negative follow-up time were excluded.

The study was approved by the Swedish Ethical Review Authority (approval numbers 2025-08088-01 and 2020-00715). All data were pseudonymized before analysis, and no patients were contacted. Individual informed consent was waived in the approval, as is standard for register-based research in Sweden (The Ethical Review Act, law 2003:460).

### Exposure

Exposure was the first dispensed prescription of spironolactone or eplerenone during the enrolment window. Treatment persistence, dose, and switching to the alternate MRA during follow-up were ascertained from subsequent dispensing records.

### Outcomes

#### Effectiveness outcomes

Four effectiveness outcomes were defined: (i) all-cause mortality; (ii) CV mortality, defined as death with an underlying cause coded I00–I99 in the Swedish Cause of Death Register; (iii) HF hospitalization, defined as an inpatient admission with a primary HF diagnosis (codes as above); and (iv) a composite of CV mortality (death with I00-99 codes) and HF hospitalization.

#### Safety outcomes

Three kidney outcomes were defined. The first was a decline in estimated glomerular filtration rate (eGFR) of more than 50% from baseline. The second was sustained eGFR <30 mL/min/1.73 m^2^, defined as two consecutive eGFR values below 30 separated by at least 90 days, and restricted to patients with a baseline eGFR ≥30. The third was a composite kidney outcome, defined as any of: a > 50% decline in eGFR, sustained eGFR <15 mL/min/1.73 m^2^ (two values at least 90 days apart), or initiation of dialysis (ICD-10 Z49.1, Z49.2, Z99.2). The 50% decline and the <15 mL/min/1.73 m^2^ threshold for the composite reflect endpoints used in landmark trials of chronic kidney disease.^17^

Estimated glomerular filtration rate was calculated from each serum creatinine measurement using the revised Lund-Malmö formula, which has been validated in Swedish populations^18^ and shown to be accurate in HF.^19,20^ Baseline eGFR was derived from the creatinine measurement closest to, but no later than, the index date, within 365 days.

Hyperkalaemia was assessed at three thresholds: serum potassium ≥5.5, ≥6.0, and ≥6.5 mmol/L. This analysis used post-index laboratory measurements and was restricted to patients with at least one potassium measurement during follow-up (*n* = 10 240).

#### Negative control outcome

A composite negative control outcome was defined as the first occurrence of cancer death, accidental death, or hip fracture surgery. No plausible mechanism links MRA selectivity to these events. A hazard ratio (HR) close to 1 would support the validity of the analytical approach.

### Covariates

Covariates were ascertained from routinely recorded administrative and laboratory data. Diagnoses from the National Patient Register (ICD-10 codes recorded by the treating physician at inpatient or outpatient contacts), dispensed medications from the Prescribed Drug Register (ATC codes), and laboratory values from the three regional clinical laboratories. Complete operational definitions for every covariate, outcome, and exclusion criterion are provided in Supplementary material online, *Table S1*. Covariates were grouped into five domains, and all were defined before the index date.

*Demographics:* age at HF diagnosis, sex, and calendar year of the index date.

*Comorbidities:* identified from any inpatient or outpatient contact before the HF diagnosis: hypertension, chronic obstructive pulmonary disease, atrial fibrillation or flutter, ischaemic heart disease, valvular heart disease, obesity, diabetes mellitus (any type), prior stroke or transitoric ischaemic attack (TIA), prior cancer within 5 years, and myocardial infarction (MI) within 12 months of the index date.

*Heart failure medications dispensed during the 12 months before the index date:* loop diuretics, beta-blockers, angiotensin-converting enzyme inhibitors (ACEIs), angiotensin receptor blockers (ARBs), angiotensin receptor–neprilysin inhibitors (ARNIs), and digoxin.

*Baseline laboratory values*, defined as the measurement closest to the index date within 365 days: N-terminal pro-B-type natriuretic peptide (NT-proBNP), serum creatinine, sodium, potassium, and haemoglobin.

*Prior hospitalizations* in the 12 months before the index date: Heart failure hospitalization and all-cause hospitalization. The 30 days immediately preceding the index date were excluded from this count to avoid capturing the index HF episode.

### Statistical analysis

#### Main analysis

Baseline characteristics were summarized by treatment group and compared using standardized mean differences (SMDs). Time-to-event outcomes were analysed using multivariable Cox proportional hazards regression. The primary models were adjusted for age, sex, calendar year of the index date, comorbidities, HF medications at baseline and prior HF hospitalizations. For the kidney outcomes, baseline eGFR was added as a covariate, restricting these models to patients with a baseline eGFR measurement. Similarly, models of hyperkalaemia outcomes were restricted to patients with a baseline potassium. Each model was fitted on complete cases for its included covariates. Direct-adjusted cumulative incidence curves were generated by predicting the cumulative incidence under each treatment assignment for every patient, then averaging across the cohort.

#### Sensitivity

Five different sensitivity analyses were performed.

First, a competing-risk analysis was conducted for HF hospitalization. A Fine–Gray subdistribution hazard model was fitted with all-cause death as the competing event. Laboratory covariates were omitted from this model to retain the full analysable cohort.

Second, propensity score matching was performed. A logistic regression model estimated the probability of receiving eplerenone, conditional on all baseline covariates and laboratory values. Patients were matched 1:3 (eplerenone:spironolactone) using nearest-neighbour matching without replacement, with a calliper of 0.2 standard deviations of the logit propensity score. Covariate balance after matching was assessed by SMD.

Third, an era-restricted analysis was conducted, in which the Cox model was repeated in patients with an index date in 2014 or later. This period corresponds to broader eplerenone use in Sweden and reduces confounding from temporal changes in background HF therapy.

Fourth, a 3-year censored analysis was conducted. The Cox model was repeated with administrative censoring at 1095 days of follow-up. This assessed whether shorter-term effects differed from those observed over the full follow-up period.

Fifth, to assess baseline balance in HF severity between treatment groups, we created a ‘cardiac severity index’. The index summed eight baseline cardiac markers ascertained at or before the index date: prior MI (within 12 months), prior HF hospitalization, hypertension, atrial fibrillation/flutter, ischaemic heart disease, presence of a cardiac implantable electronic device, digoxin use, and loop diuretic use. Non-cardiac prognostic markers were deliberately excluded. The severity index was dichotomized at the median, and the Cox model for all-cause death was then applied to both the lower and higher severity tiers to examine whether the mortality association was consistent across severity tiers.

#### Subgroups

The main Cox models were repeated in a subgroup enriched for reduced ejection fraction. Because ejection fraction is not recorded in the registers, we used a validated prescription-based proxy. A validation study in similar Danish registers demonstrated that a HF diagnosis combined with dispensing of both a renin–angiotensin system inhibitor and a beta-blocker within 120 days identifies HF with reduced ejection fraction (≤40%) with a positive predictive value of 95% (sensitivity and specificity both 85%).^21^

Additionally, the Cox models were fitted in subgroups stratified by sex (male/female), age (older or younger than 75 years), diabetes, previous MI, and baseline eGFR (≥60, 45–59, and <45 mL/min/1.73 m^2^).

#### Treatment patterns

Dispensed MRA dose was characterized by tablet strength at the index date and at ∼1 and 5 years of follow-up. Cross-over was quantified by identifying any dispensing of the alternate MRA after the index date. The proportion of switchers, the time to first switch, and the direction of switching were summarized by treatment group. Persistence at 1 and 5 years was defined as at least one dispensing within a specified window: 9–15 months for the 1-year assessment and 54–66 months for the 5-year assessment. Persistence was assessed for MRA and for the other guideline-directed HF therapies listed under covariates.

We classified MRA switching, discontinuation (no same-drug dispensing in days 183–365), and dose change (escalation/reduction vs. stable) in a 1-year landmark analysis (patients alive/event-free at 365 days). We then modelled five outcomes from the landmark using adjusted Cox regression.

#### Absolute event rates at 5 years

Predicted 5-year cumulative incidences were obtained by direct adjustment from the multivariable models. For each patient, the cumulative incidence was estimated under both treatment assignments and then averaged across the cohort, yielding a covariate-balanced absolute risk for each agent.

#### Software

All analyses were performed using R version 4.5.3 (R Foundation for Statistical Computing).

## Results

### Study population

Of 75 565 patients with a first HF diagnosis, 12 046 met the inclusion criteria and no exclusion criteria (*Figure 1*). 11 039 (91.6%) initiated spironolactone and 1007 (8.4%) initiated eplerenone. All 12 046 patients were included in the efficacy outcome models (all-cause death, CV death, HF hospitalization, and the composite of HF hospitalization and CV death). 10 927 had a baseline eGFR measurement and were included in the kidney outcome models. 10 240 had a baseline potassium measurement and were included in the hyperkalaemia models. Event rates and median follow-up times are presented in Supplementary material online, *Table S2*.

**Figure 1:**
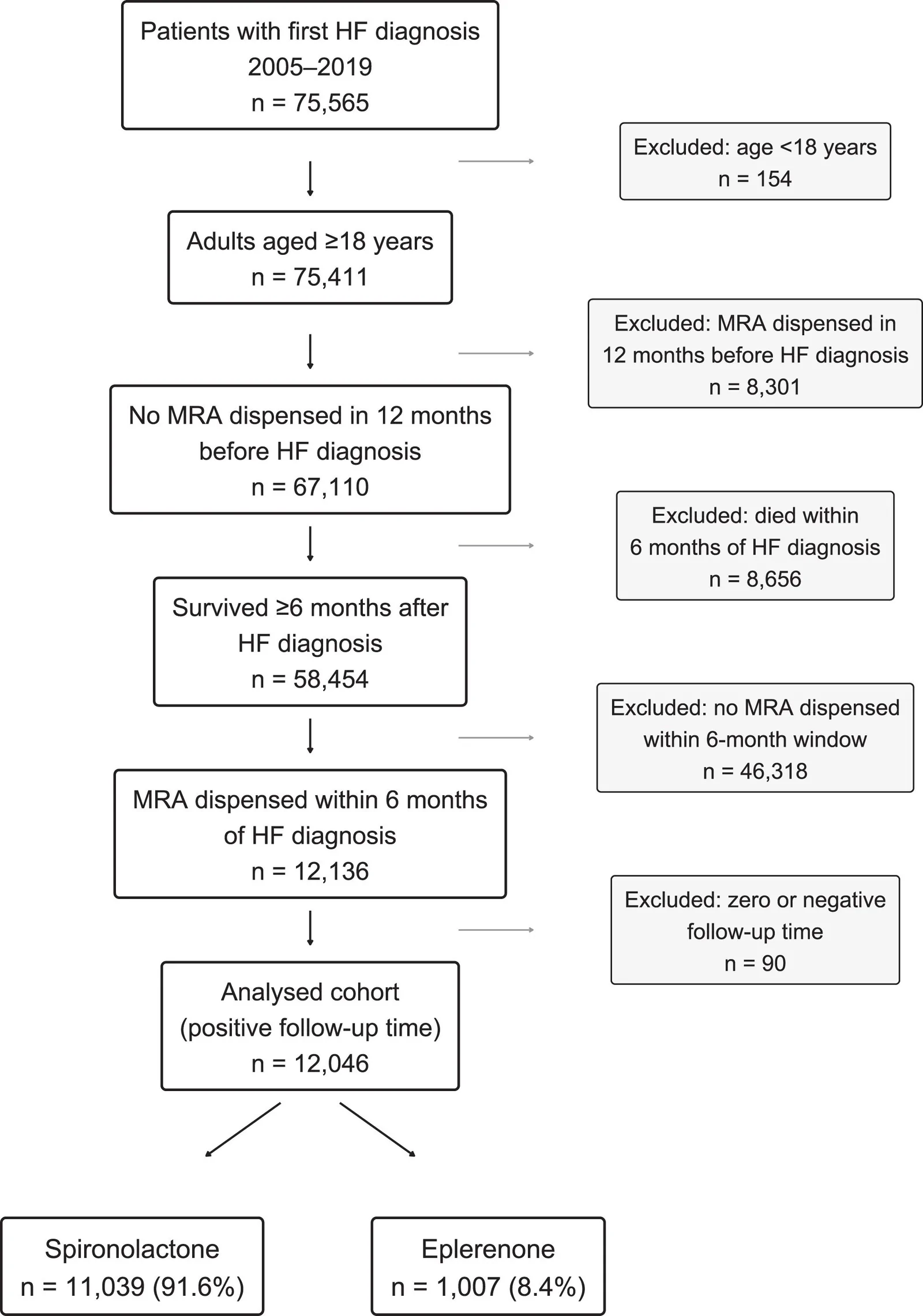
Study flow diagram. Cohort derivation. The base population was all adult residents of Stockholm County in the Stockholm Sodium Cohort with a first heart failure (HF) diagnosis (ICD-10 I50, I11.0, I13.0, I13.2, or I42.6 as a primary diagnosis) recorded between 2005 and 2019. Sequential exclusions are shown on the right. The analysed cohort comprised patients who filled a first prescription for a mineralocorticoid receptor antagonist (MRA), spironolactone or eplerenone, within 6 months of the heart failure diagnosis and contributed positive follow-up time.

### Baseline characteristics

Baseline characteristics and completeness of baseline laboratory measurement are shown in *Table 1*.

**Table 1:** Baseline characteristics of the analysis cohort, by initiated mineralocorticoid receptor antagonist

		Treatment group		
Characteristics	Overall *n* = 12,046^a^	Spironolactone *n* = 11,039^a^	Eplerenone *n* = 1,007^a^	SMD^b^
Age (years)	77 (67–84)	77 (68–84)	70 (59–78)	0.524
Sex				0.499
Male	6925 (57%)	6136 (56%)	789 (78%)	0.499
Female	5121 (43%)	4903 (44%)	218 (22%)	0.499
NT-proBNP (ng/L)	2680 (1210–5640)	2720 (1240–5734)	1980 (822–4620)	0.218
Missing	4780	4326	454	0.218
eGFR, mL/min/1.73 m^2^	60 (49–72)	60 (48–72)	68 (57–79)	0.431
Missing	1119	1066	53	0.431
Creatinine (µmol/L)	88 (73–106)	88 (73–107)	87 (73–102)	0.079
Missing	1916	1608	308	0.079
Sodium (mmol/L)	140 (138–142)	140 (138–142)	140 (139–142)	0.134
Missing	1846	1601	245	0.134
Potassium (mmol/L)	4.0 (3.7–4.3)	4.0 (3.7–4.3)	4.1 (3.8–4.4)	0.152
Missing	1749	1509	240	0.152
Haemoglobin (g/L)	131 (119–144)	131 (119–144)	140 (125–150)	0.327
Missing	2023	1700	323	0.327
Hypertension	6620 (55%)	6113 (55%)	507 (50%)	0.101
COPD	2014 (17%)	1872 (17%)	142 (14%)	0.079
Atrial fibrillation/flutter	4977 (41%)	4594 (42%)	383 (38%)	0.073
Ischaemic heart disease	4003 (33%)	3719 (34%)	284 (28%)	0.119
Valvular heart disease	1339 (11%)	1247 (11%)	92 (9.1%)	0.071
Obesity	679 (5.6%)	610 (5.5%)	69 (6.9%)	0.055
Cancer (within 5 years)	2019 (17%)	1853 (17%)	166 (16%)	0.008
Diabetes mellitus	2656 (22%)	2484 (23%)	172 (17%)	0.136
Stroke/cerebrovascular disease	1691 (14%)	1594 (14%)	97 (9.6%)	0.148
MI within 12 months of index	2351 (20%)	2157 (20%)	194 (19%)	0.007
Loop diuretic	7072 (59%)	6569 (60%)	503 (50%)	0.193
Beta-blocker	8348 (69%)	7607 (69%)	741 (74%)	0.103
ACEi/ARB/ARNI	8167 (68%)	7371 (67%)	796 (79%)	0.279
Digoxin	1396 (12%)	1294 (12%)	102 (10%)	0.051
HF hospitalization (prior 12 months)	2894 (24%)	2662 (24%)	232 (23%)	0.025
Any hospitalization (prior 12 months)	6649 (55%)	6117 (55%)	532 (53%)	0.052

Continuous variables are presented as medians (interquartile ranges) and categorical variables as numbers (percentages), each calculated on complete cases for that variable. Standardized mean differences (SMD) quantify the between-group imbalance for each characteristic; values >0.10 are conventionally regarded as meaningfully imbalanced. Comorbidities were identified from any inpatient or outpatient contact recorded before the heart failure (HF) diagnosis. Cancer was restricted to the 5 years preceding diagnosis; myocardial infarction was restricted to the 12 months before the index date. Concomitant medications were those dispensed in the 12 months before the index date. Prior hospitalizations excluded the 30 days immediately preceding the index date to avoid capturing the index HF episode.

ACEi, angiotensin converting enzyme inhibitor; ARB, angiotensin receptor blocker; ARNI, angiotensin receptor–neprilysin inhibitor; COPD, chronic obstructive pulmonary disease; eGFR, estimated glomerular filtration rate; MI, myocardial infarction; NT-proBNP, N-terminal pro-B-type natriuretic peptide.

^a^ *n* (%); median (Q1–Q3).

^b^SMD, standardized mean difference.

Patients initiating eplerenone were younger (median age 70 vs. 77 years) and more often male (78% vs. 56%). Baseline eGFR was higher in the eplerenone group (median 68 vs. 60 mL/min/1.73 m^2^) and baseline NT-proBNP was lower (median 1980 vs. 2720 ng/L, Supplementary material online, *Figure S1*). The burden of comorbidities was lower in the eplerenone group, including diabetes, atrial fibrillation, and cerebrovascular disease. The prevalence of MI within 12 months of the index date was similar (19% vs. 20%).

Concurrent guideline-directed HF therapy also differed. Patients initiating eplerenone were more often co-dispensed ACEis, ARBs, or ARNIs (79% vs. 67%) and beta-blockers (74% vs. 69%). Loop diuretic use was less frequent in the eplerenone group (50% vs. 60%).

### Main analysis

Direct-adjusted cumulative incidence curves for one outcome from each domain are shown in *Figure 2*. Hazard ratios from all outcomes are shown in *Figure 3*.

**Figure 2:**
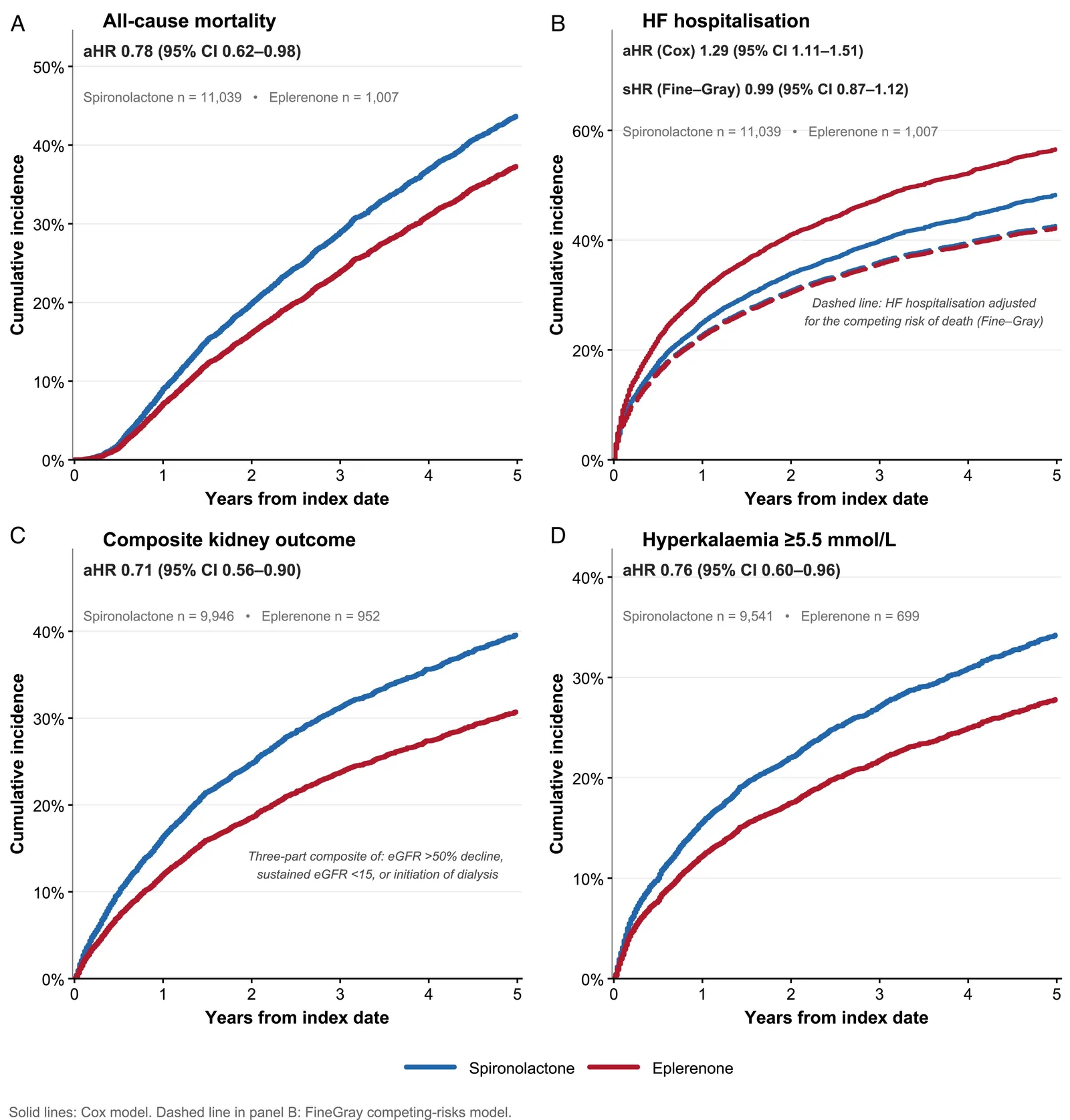
Direct-adjusted cumulative incidence curves over 5 years for four selected outcomes. (*A*) All-cause mortality. (*B*) Heart failure (HF) hospitalization, with the cause-specific Cox model (solid lines) and the Fine–Gray subdistribution model with all-cause death as the competing event (dashed lines). (*C*) Composite kidney outcome (any of: >50% decline in estimated glomerular filtration rate (eGFR), sustained estimated glomerular filtration rate <15 mL/min/1.73 m^2^, or initiation of dialysis). (*D*) Hyperkalaemia ≥5.5 mmol/L. Curves were generated by direct adjustment: for each patient, the cumulative incidence was predicted under each treatment assignment and then averaged across the cohort. The adjusted hazard ratio (aHR) from the cause-specific Cox model is annotated in each panel; (*B*) additionally reports the subdistribution hazard ratio (sHR) from the Fine–Gray model.

**Figure 3:**
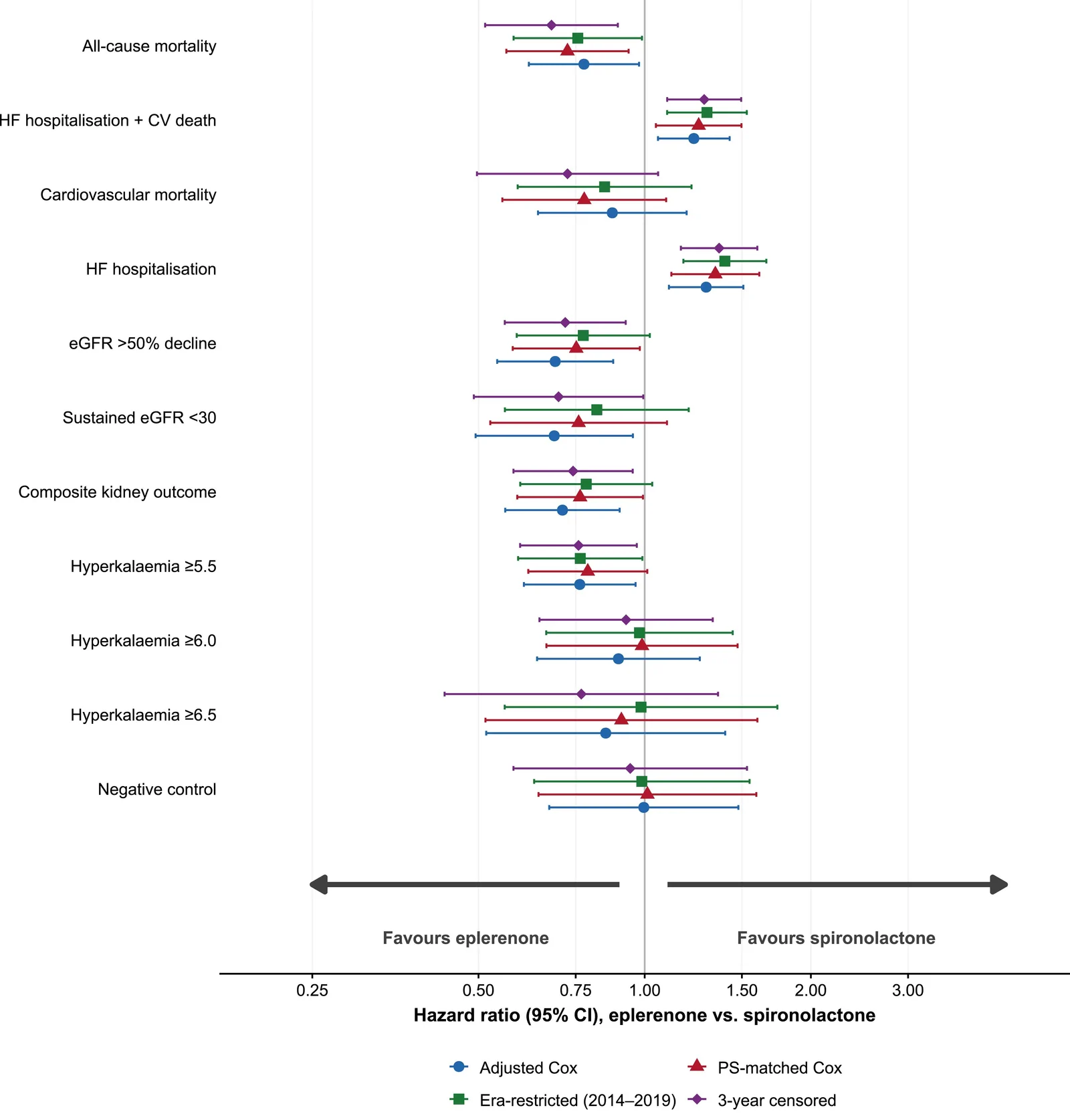
Hazard ratios for all 11 outcomes across four analytical approaches. Adjusted hazard ratios (aHR) and 95% confidence intervals (CI) for eplerenone vs. spironolactone for each outcome, displayed under four analytical approaches: the main multivariable Cox model (adjusted Cox); a 1:3 propensity score-matched Cox model; a Cox model restricted to patients with an index date in 2014 or later (era-restricted); a Cox model with administrative censoring at 3 years (3-year censored). The vertical line at hazard ratio = 1 indicates no difference between agents; estimates to the left favour eplerenone; estimates to the right favour spironolactone.

#### All-cause mortality

Eplerenone was associated with lower all-cause mortality compared with spironolactone [adjusted hazard ratio (aHR) 0.78, 95% confidence interval (CI) 0.62–0.98; *P* = 0.031; *Figure 2A*].

#### Cardiovascular mortality

No significant difference in CV mortality was observed between groups (aHR 0.87, 95% CI 0.64–1.19; *P* = 0.393).

#### Heart failure hospitalization

In the main Cox analysis, eplerenone was associated with a higher cause-specific rate of HF hospitalization (aHR 1.29, 95% CI 1.11–1.51, *P* = 0.001). In the Fine–Gray competing-risk analysis with all-cause death as the competing event, the subdistribution HR (sHR) was attenuated to sHR 0.99 (95% CI 0.87–1.12; *Figure 2B*).

#### Composite of cardiovascular death and heart failure hospitalization

Eplerenone was associated with a higher rate of the composite outcome (aHR 1.23, 95% CI 1.06–1.43; *P* = 0.007). The composite estimate was largely driven by the HF hospitalization component.

#### Kidney outcomes

Eplerenone was associated with a lower risk of >50% eGFR decline from baseline (aHR 0.69, 95% CI 0.54–0.88, *P* = 0.002), lower risk of sustained eGFR <30 mL/min/1.73 m^2^ (aHR 0.69, 95% CI 0.49–0.95; *P* = 0.024), as well as a lower risk of the composite kidney outcome (aHR 0.71, 95% CI 0.56–0.90, *P* = 0.005; *Figure 2C*).

#### Hyperkalaemia

Eplerenone was associated with a lower risk of hyperkalaemia ≥5.5 mmol/L (aHR 0.76, 95% CI 0.60–0.96, *P* = 0.023; *Figure 2D*). At higher thresholds, point estimates also favoured eplerenone, but CIs included 1: ≥6.0 mmol/L (aHR 0.90, 95% CI 0.64–1.26) and ≥6.5 mmol/L (aHR 0.85, 95% CI 0.52–1.40).

#### Negative control outcome

The negative control outcome did not differ between groups (aHR 1.00, 95% CI 0.67–1.48; *P* = 0.986).

### Absolute event rates

For all-cause mortality, the predicted 5-year incidence was 37.1% with eplerenone vs. 43.5% with spironolactone. The composite kidney outcome occurred in 30.8% vs. 39.7%, and >50% eGFR decline in 29.8% vs. 39.4%. Hyperkalaemia ≥5.5 mmol/L was lower with eplerenone (27.8%) than with spironolactone (34.3%). For HF hospitalization, the cause-specific model predicted a higher incidence with eplerenone (56.6%) than with spironolactone (48.3%), whereas in the Fine–Gray model accounting for the competing risk of death the two were almost identical (42.3% vs. 42.7%).

### Sensitivity analyses

The propensity score-matched cohort (*n* = 2027) was well balanced after matching (SMDs <0.1 for all covariates, Supplementary material online, *Figure S2*). Results from the propensity score-matched cohort, the era-restricted cohort (*n* = 5778), and the 3-year censored analysis were consistent with the main analysis. Results from the sensitivity analyses are shown in *Figure 3*.

The negative control outcome was close to 1 in both the main Cox model and the sensitivity analyses shown in *Figure 3* (all *P* > 0.8).

The mean cumulative number of laboratory measurements of creatinine and potassium following the index date were approximately similar over time. See Supplementary material online, *Figure S3*.

The distribution of the cardiac severity index was approximately similar between groups. See supplementary material online, *Figure S4*. The aHR for all-cause mortality was 0.69 (95% CI 0.48–1.00) in the lower tier, and 0.83 (95% CI 0.62–1.11) in the higher tier; interaction *P* = 0.42. See Supplementary material online, *Figure S5*.

### Subgroup analyses

For all-cause mortality, point estimates favoured eplerenone in every subgroup defined by age, sex, diabetes, and MI within 12 months, with no significant interaction on the multiplicative scale (all interaction *P* ≥ 0.10; Supplementary material online, *Figure S6*). When outcomes were stratified by baseline eGFR (≥60, 45–59, and <45 mL/min/1.73 m^2^; Supplementary material online, *Figure S7*), the kidney estimates were precise only in the eGFR ≥60 stratum, where both >50% eGFR decline and the composite kidney outcome had an aHR of 0.56 (95% CI 0.39–0.79).

In the subgroup enriched for patients with reduced ejection fraction (*n* = 8646), the aHR for all-cause mortality attenuated to 0.85 and the CI included 1 (95% CI 0.65–1.10). Adjusted hazard ratio for the composite kidney outcome was 0.73 (95% CI 0.57–0.95), and aHR for hyperkalaemia ≥5.5 mmol/L was 0.76 (95% CI 0.59–0.98). Full results are shown in Supplementary material online, *Figure S8*.

### Treatment switching

Switching (6.0% within the first year) was not associated with higher mortality (aHR 0.80, 0.63–1.00) or worse cardiovascular, kidney, or hyperkalaemia outcomes. Discontinuation was associated with higher mortality and HF hospitalization, but fewer kidney and hyperkalaemia events. Dose change in either direction was associated with higher mortality. Results are presented in Supplementary material online, *Table S3*. Persistence patterns are presented in Supplementary material online, *Figure S9*.

## Discussion

In this cohort of patients with newly diagnosed HF, initiation of eplerenone was associated with lower all-cause mortality and more favourable kidney outcomes compared with initiation of spironolactone. To our knowledge, this is the first study to report long-term kidney and electrolyte safety outcomes using individual-level laboratory data.

### All-cause and cardiovascular mortality

Eplerenone was associated with a 22% lower hazard of all-cause mortality, closely matching a recent nationwide Danish emulation^22^ and a recent systematic review.^15^ Our cohort did not include ejection fraction, so residual confounding by HF phenotype remains possible. The similar finding in the Danish study, conducted in patients with echocardiographically confirmed HF with reduced ejection fraction, provides partial reassurance against confounding by phenotype. The finding was also robust to propensity score matching, era-restriction, and 3-year censoring and was supported by the null negative control outcome.

Cardiovascular mortality did not differ significantly between groups, although misclassification of the underlying cause of death, previously described in Swedish HF registries, may attenuate this estimate.^23,24^

The mechanism underlying the potential mortality difference is uncertain. Eplerenone has been associated with more favourable myocardial remodelling in small, randomized trials, and a recent meta-analysis.^10–12^ Differences in selectivity, tolerability, and persistence may also contribute. In our data, MRA persistence was higher with eplerenone at both 1 and 5 years, while persistence for other guideline-directed HF therapies largely did not differ (see Supplementary material online, *Figure S9*). This is consistent with the Danish emulation, in which the mortality advantage attenuated in per-protocol analyses and was likewise attributed partly to better persistence,^22^ and with an earlier Danish cohort in which treatment withdrawal was more frequent with spironolactone.^14^ The more favourable kidney profile with eplerenone may also contribute.

In the subgroup enriched for reduced ejection fraction, the mortality association was directionally consistent but attenuated and no longer significant (aHR 0.85, 95% CI 0.65–1.10), while the kidney and hyperkalaemia associations were essentially unchanged. This prescription-based definition, though validated against measured ejection fraction,^21^ inevitably misclassifies some patients. The point estimate remained close to the main analysis (0.85 vs. 0.78) in a subgroup with fewer events, so we do not read the attenuation as evidence that the mortality association is confined to patients without reduced ejection fraction. Nonetheless, the loss of significance in the subgroup most likely to represent HF with reduced ejection fraction underscores that our mortality estimate should be interpreted with caution, and the comparative mortality of the two agents is best regarded as a question that observational data can support but not settle.

### Heart failure hospitalization and competing risks

The higher cause-specific hazard for HF hospitalization with eplerenone was unexpected. It is not in line with prior comparative data or with the pharmacological hypothesis. In the Fine–Gray analysis, with all-cause death as the competing event, this difference disappeared. This attenuation is consistent with the survival difference between groups: patients in the eplerenone group lived longer and therefore had more opportunity to be hospitalized. The subdistribution HR is the more clinically meaningful estimate, as it accounts for the competing risk of death and reflects the absolute hospitalization risk experienced by patients. The neutral subdistribution hazard we observed is also in accordance with the neutral estimate for HF hospitalization in the Danish emulated trial (weighted HR 0.97, 95% CI 0.87–1.09).^22^

### Kidney outcomes and hyperkalaemia

Eplerenone was associated with a 31% lower risk of both >50% eGFR decline and sustained eGFR <30 mL/min/1.73 m^2^. These associations were robust across sensitivity analyses. The lower risk of hyperkalaemia ≥5.5 mmol/L with eplerenone is consistent with the pharmacological expectation that eplerenone has a shorter half-life and fewer active metabolites. Point estimates at the ≥6.0 and ≥6.5 mmol/L thresholds were directionally consistent but with wider CIs, reflecting fewer events at higher thresholds.

A 50% decrease in eGFR could at least partially reflect an expected haemodynamic effect of MRA rather than intrinsic kidney damage. However, the consistent signal across different kidney outcomes, including sustained declines in eGFR, speaks to the clinical relevance of findings.

The eplerenone advantage for kidney function preservation was more clearly observed in patients with preserved baseline kidney function (eGFR ≥60 mL/min/1.73 m^2^). In patients with reduced baseline kidney function, sample sizes were smaller, and CIs were correspondingly wide. Additionally, the negative control outcome favoured spironolactone in the eGFR 45–59 mL/min/1.73 m^2^ group, suggesting residual confounding.

### Treatment switching

Switching between MRAs did not worsen outcomes, whereas discontinuation and dose changes marked higher-risk patients. This likely reflects confounding by indication rather than direct effects, and supports the robustness of our intention-to-treat estimates.

### Strengths and limitations

This study has several strengths. The source cohort is defined by serum or plasma sodium measurement, a near-universal investigation in HF, so patients enter the cohort largely independently of the clinic or healthcare setting in which they are managed, reducing the selection bias that affects cohorts recruited from specialist clinics. Availability of individual-level laboratory data enabled direct assessment of kidney and electrolyte safety outcomes that have not previously been reported in comparative studies of these agents. Follow-up extended well beyond the 2-year limit of most prior work, allowing sufficient time for kidney events to accumulate. The inclusion of a negative control outcome provides reassurance against residual confounding, and the consistency of findings across multiple sensitivity analyses strengthens confidence in the results.

Several limitations should be noted. First, baseline characteristics differed substantially between groups: patients initiated on eplerenone were younger, more often male, and had a lower burden of comorbidity. A similar pattern was observed in a Danish cohort^14^ and is consistent with channelling bias. In our study, this was addressed with multivariable adjustment, a negative control outcome and several sensitivity analyses. However, due to the retrospective nature of the study, residual confounding cannot be excluded.

Second, we lacked data on left ventricular ejection fraction and New York Heart Association (NYHA) functional class, because standardized phenotype codes were introduced in Sweden in 2021. The sensitivity analyses partially address this, but confounding by HF phenotype remains a possibility, particularly for the mortality outcome.

Third, eplerenone was disproportionately prescribed in later calendar years, during which overall HF management improved. Adjustment for index year and the era-restricted sensitivity analysis mitigate this concern but do not eliminate it.

Fourth, all outcomes and covariates were ascertained from data recorded during routine clinical care rather than for research purposes, and coding practices may vary between clinicians and over time. However, the Swedish registers used to build the SSC are extensively validated.^16^ Additionally, the exposure and the kidney and potassium outcomes derive from objective dispensing and laboratory records rather than diagnostic coding. Non-differential misclassification would be expected to bias estimates towards the null.

### Clinical implications

European Society of Cardiology guidelines give MRAs a Class I recommendation in HF with reduced ejection fraction but express no preference between eplerenone and spironolactone, and the two agents have not been compared head-to-head in a randomized trial.^6,7^ Our findings, together with other recent literature, suggest that the choice between the two may not be clinically inconsequential. The observed differences in kidney function decline, hyperkalaemia, and treatment persistence may be relevant when selecting an MRA for an individual patient. However, given the observational design, the baseline differences between groups, and the absence of ejection fraction data, our findings should not be taken to establish the superiority of one agent over the other across the HF population.

## Conclusions

In this cohort with newly diagnosed HF, initiation of eplerenone rather than spironolactone was associated with lower all-cause mortality and lower risks of adverse kidney outcomes and mild hyperkalaemia. These associations persisted across multiple sensitivity analyses, but residual confounding cannot be excluded and confirmation in randomized comparisons is required.

## Supplementary Material

pvag064_Supplementary_Data

## Data Availability

The data underlying this study were obtained from Swedish national health registries under approval from the Swedish Ethical Review Authority (2025-08088-01 and 2020-00715). Per Swedish law and the conditions of the ethical approval, individual-level data cannot be shared publicly. Analysis code is available from the corresponding author upon reasonable request.

## Appendices

### Treatment patterns

#### Dose

The 25 mg tablet predominated in both groups (90% of spironolactone users and 84% of eplerenone users). The 50 mg dose was used by 8% and 14%, respectively. Fewer than 1% of patients in either group received 100 mg (see Supplementary material online, *Table S4*).

#### Persistence

Persistence was higher in the eplerenone group at both time points: 73.4% vs. 63.3% at 1 year and 55.1% vs. 45.5% at 5 years. Among persistent users, the dose distribution remained stable over time.

#### Cross-over

During follow-up, 5.8% of spironolactone users and 7.7% of eplerenone users were dispensed the alternate MRA at least once. Median time to first cross-over was 393 days from spironolactone to eplerenone and 329 days from eplerenone to spironolactone.

#### Prescription patterns over time

Eplerenone prescriptions increased incrementally during the study period, whereas spironolactone prescriptions remained stable (see Supplementary material online, *Figure S10*).
