# Supplementary material for: Eplerenone vs. spironolactone in heart failure: long-term cardiorenal and mortality outcomes in a registry-based cohort
Source: Eur Heart J Cardiovasc Pharmacother. 2026 Sep 10;12(7):501–11. doi: 10.1093/ehjcvp/pvag064 (PMC13730579; doi:10.1093/ehjcvp/pvag064)

Figure S5. All-cause mortality by HF-severity half

Median split of eight-component index

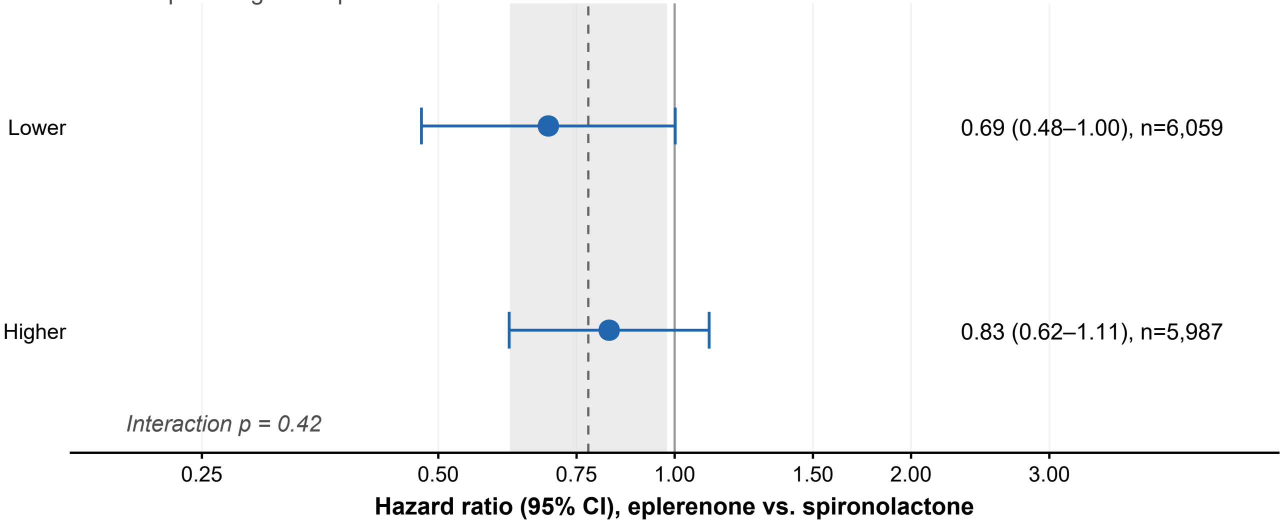

Supplement: Supplementary file 1 [file pvag064_supplementary_data.zip › FigureS5_severity_mortality_forest.pdf]
