# Supplementary material for: Eplerenone vs. spironolactone in heart failure: long-term cardiorenal and mortality outcomes in a registry-based cohort
Source: Eur Heart J Cardiovasc Pharmacother. 2026 Sep 10;12(7):501–11. doi: 10.1093/ehjcvp/pvag064 (PMC13730579; doi:10.1093/ehjcvp/pvag064)

Figure S6. All-cause mortality by clinical subgroup

Eplerenone vs. spironolactone; grey band = overall adjusted HR

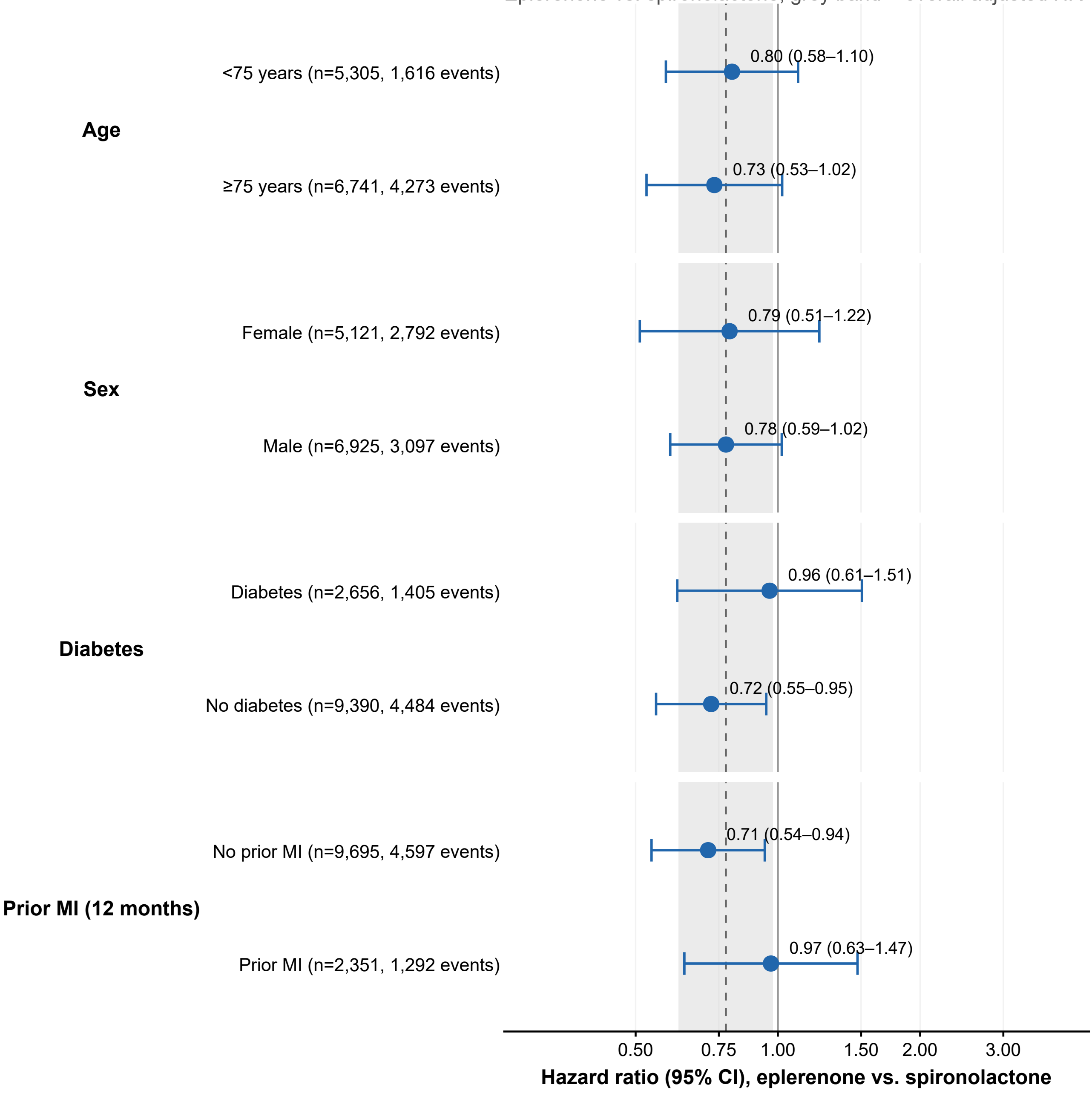

Supplement: Supplementary file 1 [file pvag064_supplementary_data.zip › FigureS6_subgroup_mortality.pdf]
