# Supplementary material for: Eplerenone vs. spironolactone in heart failure: long-term cardiorenal and mortality outcomes in a registry-based cohort
Source: Eur Heart J Cardiovasc Pharmacother. 2026 Sep 10;12(7):501–11. doi: 10.1093/ehjcvp/pvag064 (PMC13730579; doi:10.1093/ehjcvp/pvag064)
Supplement: Supplementary file 1 [file pvag064_supplementary_data.zip › FigureS7_all_outcomes_by_ckd.pdf]

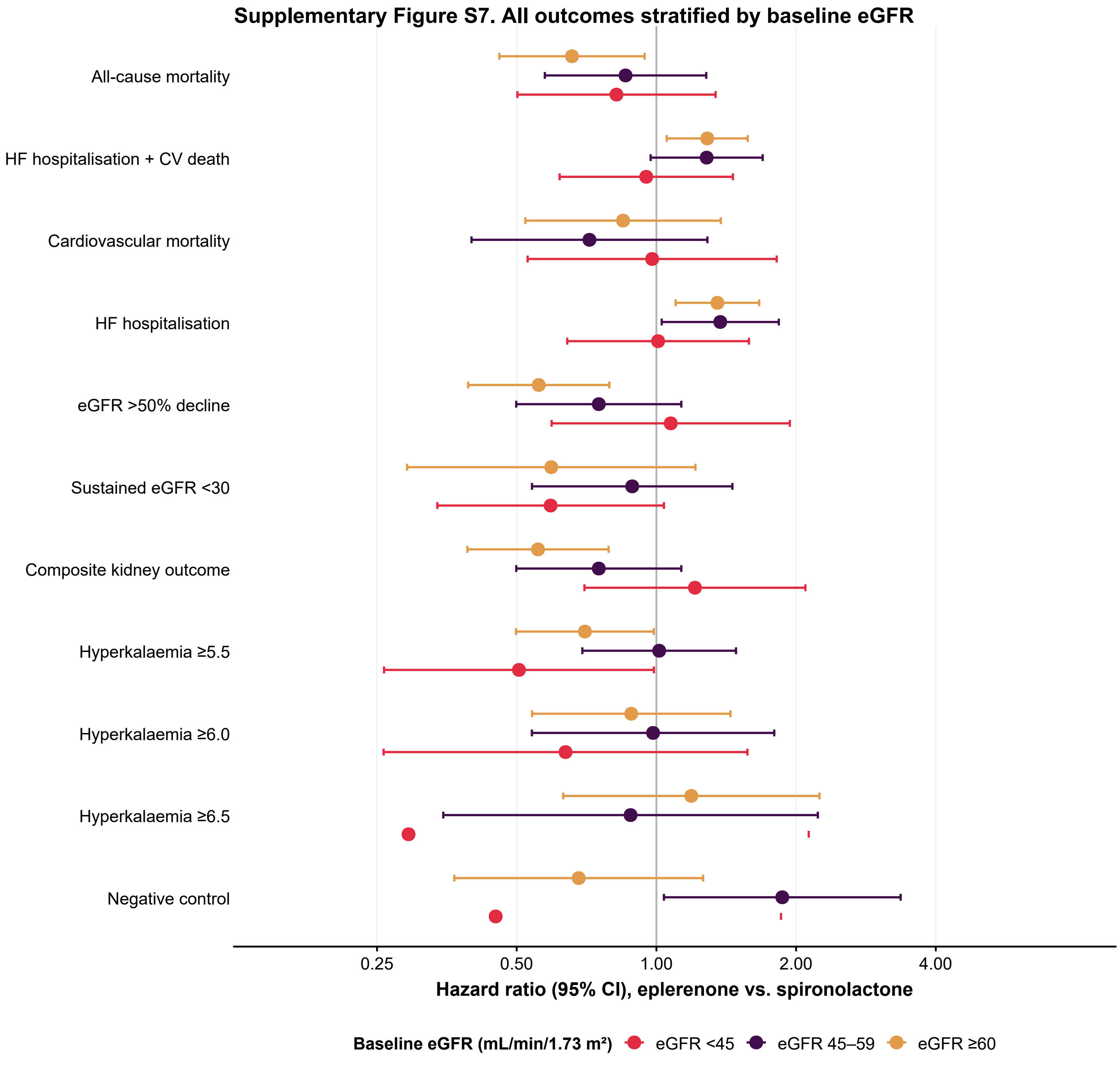

Supplementary Figure S7. All outcomes stratified by baseline eGFR
All-cause mortality
HF hospitalisation + CV death
Cardiovascular mortality
HF hospitalisation
eGFR >50% decline
Sustained eGFR <30
Composite kidney outcome
Hyperkalaemia ≥5.5
Hyperkalaemia ≥6.0
Hyperkalaemia ≥6.5
Negative control
0.25
0.50
1.00
2.00
4.00
Hazard ratio (95% CI), eplerenone vs. spironolactone
Baseline eGFR (mL/min/1.73 m²)
eGFR <45
eGFR 45–59
eGFR ≥60
