# Supplementary material for: Eplerenone vs. spironolactone in heart failure: long-term cardiorenal and mortality outcomes in a registry-based cohort
Source: Eur Heart J Cardiovasc Pharmacother. 2026 Sep 10;12(7):501–11. doi: 10.1093/ehjcvp/pvag064 (PMC13730579; doi:10.1093/ehjcvp/pvag064)
Supplement: Supplementary file 1 [file pvag064_supplementary_data.zip › FigureS8_HFrEF_enriched.pdf]

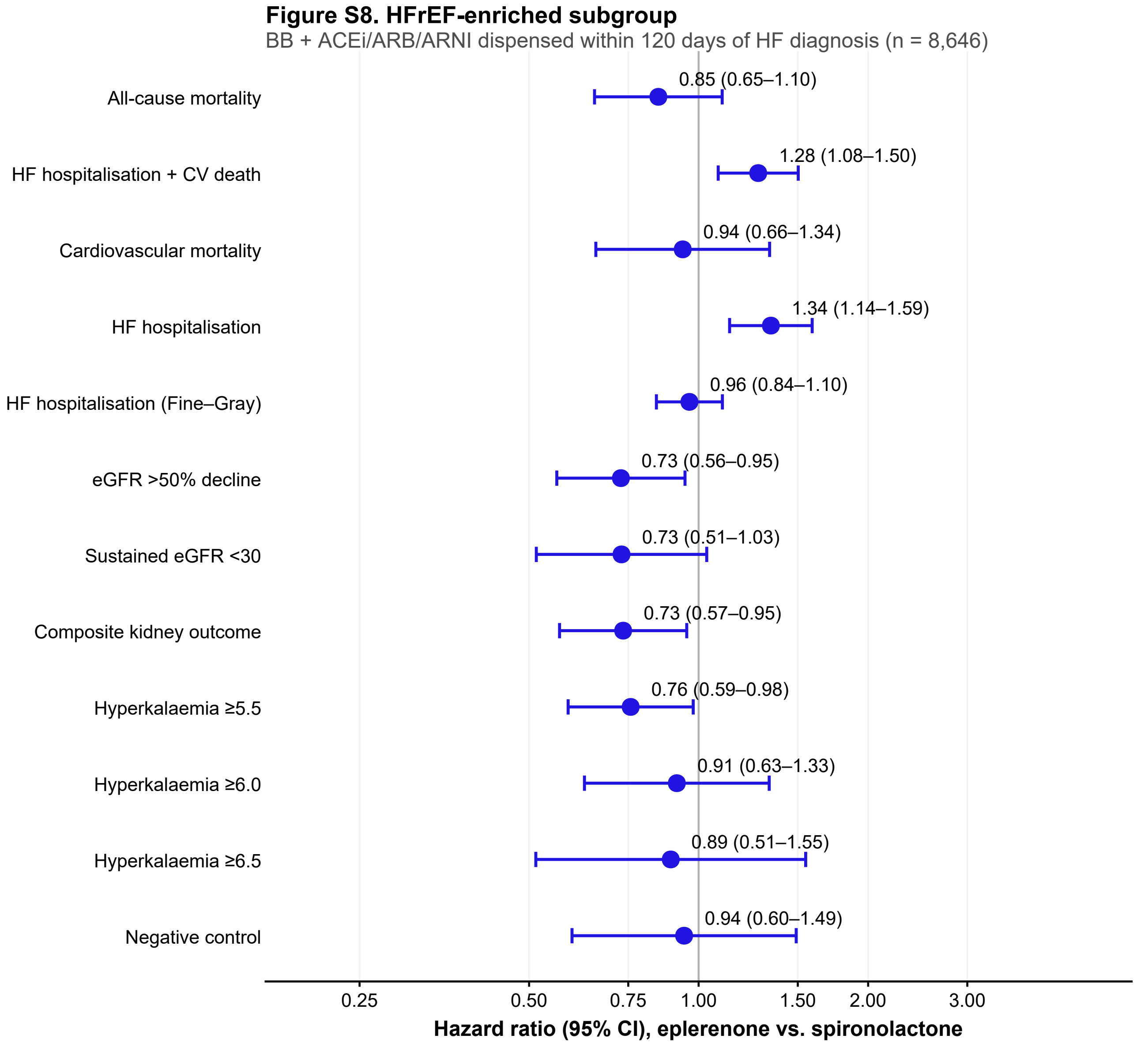
Figure S8. HFrEF-enriched subgroup
BB + ACEi/ARB/ARNI dispensed within 120 days of HF diagnosis (n = 8,646)
All-cause mortality
0.85 (0.65–1.10)
HF hospitalisation + CV death
1.28 (1.08–1.50)
Cardiovascular mortality
0.94 (0.66–1.34)
HF hospitalisation
1.34 (1.14–1.59)
HF hospitalisation (Fine–Gray)
0.96 (0.84–1.10)
eGFR >50% decline
0.73 (0.56–0.95)
Sustained eGFR <30
0.73 (0.51–1.03)
Composite kidney outcome
0.73 (0.57–0.95)
Hyperkalaemia ≥5.5
0.76 (0.59–0.98)
Hyperkalaemia ≥6.0
0.91 (0.63–1.33)
Hyperkalaemia ≥6.5
0.89 (0.51–1.55)
Negative control
0.94 (0.60–1.49)
0.25
0.50
0.75
1.00
1.50
2.00
3.00
Hazard ratio (95% CI), eplerenone vs. spironolactone
