# Supplementary material for: Eplerenone vs. spironolactone in heart failure: long-term cardiorenal and mortality outcomes in a registry-based cohort
Source: Eur Heart J Cardiovasc Pharmacother. 2026 Sep 10;12(7):501–11. doi: 10.1093/ehjcvp/pvag064 (PMC13730579; doi:10.1093/ehjcvp/pvag064)
Supplement: Supplementary file 1 [file pvag064_supplementary_data.zip › Supplementary Figures and Tables captions.docx]

**Supplementary Figure S1. Baseline NT-proBNP by treatment group.** Distribution of baseline N-terminal pro–B-type natriuretic peptide (NT-proBNP), the measurement closest to the index date within one year, by treatment group, on a logarithmic scale. Restricted to patients with an available baseline NT-proBNP (eplerenone n = 553; spironolactone n = 6,713).

**Supplementary Figure S2. Propensity score matching diagnostics.** (A) Density of the estimated propensity score before and after matching, by treatment group. (B) Absolute standardised mean differences (SMD) for each covariate in the unmatched and matched cohorts. The propensity score was estimated by logistic regression on all baseline covariates and laboratory values used in the main analysis. Matching was 1:3 (eplerenone:spironolactone) nearest-neighbour without replacement, with a caliper of 0.2 standard deviations of the logit propensity score. The vertical reference line at SMD = 0.1 indicates the conventional threshold for acceptable covariate balance.

**Supplementary Figure S3. Monitoring intensity over follow-up.** Mean cumulative number of creatinine and potassium measurements per patient by year since the index date, stratified by treatment group.

**Supplementary Figure S4. Distribution of the cardiac severity index by treatment group.** Percentage of patients in each treatment group at each value of the cardiac severity index (range 0–8), which sums eight baseline cardiac markers ascertained at or before the index date: prior myocardial infarction (within 12 months), prior heart failure hospitalisation, hypertension, atrial fibrillation/flutter, ischaemic heart disease, presence of a cardiac implantable electronic device, digoxin use, and loop diuretic use.

**Supplementary Figure S5. All-cause mortality by cardiac severity index tier.** Adjusted hazard ratios (aHR) and 95% confidence intervals for all-cause mortality with eplerenone versus spironolactone within the lower and higher halves of the cardiac severity index (dichotomised at the median). The p-value is for the multiplicative interaction between treatment and severity tier. The index is defined in Supplementary Figure S4.

**Supplementary Figure S6. All-cause mortality across subgroups.** Adjusted hazard ratios (aHR) and 95% confidence intervals for all-cause mortality with eplerenone versus spironolactone within subgroups defined by age (<75 vs ≥75 years), sex, diabetes, and myocardial infarction within 12 months of the index date, each from a separate multivariable Cox model. The grey band indicates the overall adjusted hazard ratio (95% CI) and the dashed line its point estimate.

**Supplementary Figure S7. Hazard ratios for all outcomes by baseline estimated glomerular filtration rate stratum.** Adjusted hazard ratios (aHR) and 95% confidence intervals for eplerenone versus spironolactone for all outcomes stratified by baseline eGFR (≥60, 45–59, and <45 mL/min/1.73 m²). Each stratum was analysed with a separate multivariable Cox model. The vertical line at HR = 1 indicates no difference between agents; estimates to the left favour eplerenone; estimates to the right favour spironolactone.

**Supplementary Figure S8. Hazard ratios in the subgroup enriched for reduced ejection fraction.** Adjusted hazard ratios (aHR) and 95% confidence intervals for eplerenone versus spironolactone for all 11 outcomes, restricted to patients dispensed a beta-blocker together with an angiotensin-converting-enzyme inhibitor (ACEI), angiotensin receptor blocker (ARB), or angiotensin receptor–neprilysin inhibitor (ARNI) within 120 days of the heart failure diagnosis (n = 8,646), a validated prescription-based proxy for heart failure with reduced ejection fraction. The vertical line at HR = 1 indicates no difference between agents; estimates to the left favour eplerenone, to the right favour spironolactone.

**Supplementary Figure S9. Treatment persistence of heart failure medications at one and five years.** Proportion of baseline users of each medication class with at least one dispensing during the assessment window (9–15 months for the one-year assessment; 54–66 months for the five-year assessment), restricted to patients whose follow-up extended to the start of the relevant window. Results are stratified by the initiated mineralocorticoid receptor antagonist (MRA). ACEI, angiotensin-converting-enzyme inhibitor; ARB, angiotensin receptor blocker; ARNI, angiotensin receptor–neprilysin inhibitor.

**Supplementary Figure S10. Annual prescription pattern of spironolactone and eplerenone over the study period.** Number of new mineralocorticoid receptor antagonist (MRA) initiators in the analysed cohort by calendar year of the index date, stratified by drug.

**Supplementary Table S1. Operational definitions of covariates, outcomes, and exclusion criteria.** Complete list of the ICD-10 diagnosis codes, ATC medication codes, and laboratory and procedure definitions used to ascertain each covariate, outcome, and exclusion criterion from the linked registers.

**Supplementary Table S2. Event counts, event rates, follow-up distribution, and adjusted hazard ratios for all 11 outcomes.** Event rates are expressed as events per 100 person-years in the main Cox model. Follow-up is reported as the minimum, median, and maximum, in years, of the time-to-event-or-censor distribution among patients contributing to each analysis. Adjusted hazard ratios (aHR) and 95% confidence intervals are from the main multivariable Cox model. eGFR, estimated glomerular filtration rate; HF, heart failure; CV, cardiovascular.

**Supplementary Table S3. Association of treatment switching, discontinuation, and dose change with subsequent outcomes: one-year landmark analysis.** Adjusted hazard ratios (aHR) and 95% confidence intervals for the study outcomes (all-cause mortality and the cardiovascular, kidney, and hyperkalaemia outcomes), among patients alive and event-free at 365 days, according to MRA switching, discontinuation (no same-drug dispensing in days 183–365), and dose change (escalation or reduction vs stable) during the first year. Follow-up for each model begins at the one-year landmark.

**Supplementary Table S4. Dispensed tablet strength at the index date, by initiated mineralocorticoid receptor antagonist.** Distribution of tablet strength at the index dispensing. Values are expressed as numbers (percentages), n (%), within each treatment group. "Other" includes any strength outside the 25, 50, and 100 mg dose categories, as well as patients who collected two or more different tablet strengths within six monthss
