# Supplementary material for: Eplerenone vs. spironolactone in heart failure: long-term cardiorenal and mortality outcomes in a registry-based cohort
Source: Eur Heart J Cardiovasc Pharmacother. 2026 Sep 10;12(7):501–11. doi: 10.1093/ehjcvp/pvag064 (PMC13730579; doi:10.1093/ehjcvp/pvag064)
Supplement: Supplementary file 1 [file pvag064_supplementary_data.zip › TableS1.docx]

| **Category** | **Code** | **Meaning** | **Source** |
| --- | --- | --- | --- |
| Heart failure | I50 | Heart failure | ICD-10 |
|  | I11.0 | Hypertensive heart disease with (congestive) heart failure | ICD-10 |
|  | I13.0 | Hypertensive heart and kidney disease with heart failure | ICD-10 |
|  | I13.2 | Hypertensive heart and kidney disease with both heart and kidney failure | ICD-10 |
|  | I42.6 | Endomyocardial (eosinophilic) disease | ICD-10 |
| Hypertension | I10–I15 | Hypertensive diseases | ICD-10 |
| COPD | J40–J47 | Chronic lower respiratory diseases (COPD spectrum) | ICD-10 |
| Atrial fibrillation/flutter | I48 | Atrial fibrillation and flutter | ICD-10 |
| Ischaemic heart disease | I20 | Angina pectoris | ICD-10 |
|  | I21 | Acute myocardial infarction | ICD-10 |
|  | I22 | Subsequent myocardial infarction | ICD-10 |
|  | I23 | Certain current complications following acute MI | ICD-10 |
|  | I24 | Other acute ischaemic heart diseases | ICD-10 |
|  | I25 | Chronic ischaemic heart disease | ICD-10 |
| Valvular heart disease | I05 | Rheumatic mitral valve diseases | ICD-10 |
|  | I06 | Rheumatic aortic valve diseases | ICD-10 |
|  | I07 | Rheumatic tricuspid valve diseases | ICD-10 |
|  | I08 | Multiple valve diseases | ICD-10 |
|  | I34 | Nonrheumatic mitral valve disorders | ICD-10 |
|  | I35 | Nonrheumatic aortic valve disorders | ICD-10 |
|  | I36 | Nonrheumatic tricuspid valve disorders | ICD-10 |
|  | I37 | Pulmonary valve disorders | ICD-10 |
|  | I38 | Endocarditis, valve unspecified | ICD-10 |
|  | I39 | Endocardial disorders in diseases classified elsewhere | ICD-10 |
| Obesity | E66 | Obesity | ICD-10 |
| Cancer (any, 5 y) | C00–C99 | Malignant neoplasms (any site) | ICD-10 |
| Diabetes mellitus | E10 | Type 1 diabetes mellitus | ICD-10 |
|  | E11 | Type 2 diabetes mellitus | ICD-10 |
|  | E12 | Malnutrition-related diabetes mellitus | ICD-10 |
|  | E13 | Other specified diabetes mellitus | ICD-10 |
|  | E14 | Unspecified diabetes mellitus | ICD-10 |
| Stroke / cerebrovascular disease | I60 | Subarachnoid haemorrhage | ICD-10 |
|  | I61 | Intracerebral haemorrhage | ICD-10 |
|  | I62 | Other nontraumatic intracranial haemorrhage | ICD-10 |
|  | I63 | Cerebral infarction | ICD-10 |
|  | I64 | Stroke, not specified as haemorrhage or infarction | ICD-10 |
|  | I65 | Occlusion/stenosis of precerebral arteries, not causing infarction | ICD-10 |
|  | I66 | Occlusion/stenosis of cerebral arteries, not causing infarction | ICD-10 |
|  | I67 | Other cerebrovascular diseases | ICD-10 |
|  | I68 | Cerebrovascular disorders in diseases classified elsewhere | ICD-10 |
|  | I69 | Sequelae of cerebrovascular disease | ICD-10 |
| Prior myocardial infarction (12 mo) | I21 | Acute MI | ICD-10 |
|  | I22 | Subsequent MI | ICD-10 |
|  | I25.2 | Old myocardial infarction | ICD-10 |
| Dialysis | Z49.1 | Extracorporeal dialysis (haemodialysis) encounter | ICD-10 |
|  | Z49.2 | Other dialysis encounter (e.g. peritoneal) | ICD-10 |
|  | Z99.2 | Dependence on renal dialysis | ICD-10 |
| Hyperkalaemia hospitalisation | E87.5 | Hyperkalaemia | ICD-10 |
| Cardiovascular death (underlying cause) | I00–I99 | Diseases of the circulatory system (all I-codes) | ICD-10 |
| Cancer death (negative control) | C00–C99 | Any malignant neoplasm as underlying cause of death | ICD-10 |
| Accidental death (negative control) | V01–V99 | Transport accidents | ICD-10 |
|  | W00–W99 | Other external causes of accidental injury | ICD-10 |
|  | X00–X59 | Accidents (falls, exposure, drowning, etc.) | ICD-10 |
|  | Y85–Y86 | Sequelae of accidental injury / event of undetermined intent (external cause) | ICD- |
| Hip fracture / surgery (negative control) | NFB (KVÅ) | Hip joint operations (Nordic KVÅ chapter) | Procedure code / ICD-10 |
|  | NFC (KVÅ) | Knee/lower leg operations relevant to hip context (Nordic KVÅ) | Procedure code / ICD-10 |
|  | S72 | Fracture of femur (includes hip fracture) | Procedure code / ICD-10 |
| Defibrillator-containing system (KVÅ) | FPG* | Implantation/revision of implantable cardioverter-defibrillator (incl. CRT-D) | Procedure code |
| Pacemaker family incl. CRT-P (KVÅ) | FPE* | Implantation/revision of permanent pacemaker (incl. CRT-P) | Procedure code |
| Cardiac device present (ICD-10) | Z95.0 | Presence of cardiac pacemaker / device | ICD-10 |
| MRA — spironolactone | C03DA01 | Spironolactone | ATC (Swedish drug registry) |
| MRA — eplerenone | C03DA04 | Eplerenone | ATC (Swedish drug registry) |
| Loop diuretic | C03C | Loop diuretics (e.g. furosemide, bumetanide) | ATC (Swedish drug registry) |
| Beta-blocker | C07 | Beta-blocking agents | ATC (Swedish drug registry) |
| RAS inhibitor | C09A | ACE inhibitors, plain | ATC (Swedish drug registry) |
|  | C09B | ACE inhibitors, combinations | ATC (Swedish drug registry) |
|  | C09C | Angiotensin II receptor blockers, plain | ATC (Swedish drug registry) |
|  | C09D | ARB combinations (incl. ARNI, C09DX04 = sacubitril/valsartan) | ATC (Swedish drug registry) |
| Digoxin | C01AA05 | Digoxin | ATC (Swedish drug registry) |
