# Supplementary material for: Eplerenone vs. spironolactone in heart failure: long-term cardiorenal and mortality outcomes in a registry-based cohort
Source: Eur Heart J Cardiovasc Pharmacother. 2026 Sep 10;12(7):501–11. doi: 10.1093/ehjcvp/pvag064 (PMC13730579; doi:10.1093/ehjcvp/pvag064)
Supplement: Supplementary file 1 [file pvag064_supplementary_data.zip › TableS2.docx]

|  | **Spironolactone** | | | **Eplerenone** | | | **^†^** |  |  |
| --- | --- | --- | --- | --- | --- | --- | --- | --- | --- |
| **Outcome** | **N** | **Events** | **Rate*** | **N** | **Events** | **Rate*** | **FU†** | **aHR (95% CI)** | **P** |
| All-cause mortality | 11,039 | 5,764 | 12.5 | 1,007 | 125 | 5.3 | 0.00 / 3.08 / 14.49 | 0.78 (0.62-0.98) | 0.031 |
| Cardiovascular mortality | 11,039 | 3,185 | 6.9 | 1,007 | 65 | 2.8 | 0.00 / 3.08 / 14.49 | 0.87 (0.64-1.19) | 0.393 |
| HF hospitalisation | 11,039 | 4,696 | 14.5 | 1,007 | 281 | 16.1 | 0.00 / 1.82 / 14.49 | 1.29 (1.11-1.51) | 0.001 |
| HF hospitalisation + CV death | 11,039 | 5,950 | 18.4 | 1,007 | 299 | 17.2 | 0.00 / 1.82 / 14.49 | 1.23 (1.06-1.43) | 0.007 |
| eGFR >50% decline | 9,973 | 3,183 | 10.1 | 954 | 77 | 3.9 | 0.00 / 2.12 / 14.49 | 0.69 (0.54-0.88) | 0.002 |
| Sustained eGFR <30 | 9,471 | 2,009 | 6.0 | 938 | 41 | 2.0 | 0.00 / 2.44 / 14.49 | 0.69 (0.49-0.95) | 0.024 |
| Composite kidney outcome | 9,946 | 3,215 | 10.2 | 952 | 80 | 4.1 | 0.00 / 2.11 / 14.49 | 0.71 (0.56-0.90) | 0.005 |
| Hyperkalaemia ≥5.5 | 9,541 | 2,656 | 7.3 | 699 | 91 | 5.4 | 0.00 / 2.68 / 14.49 | 0.76 (0.60-0.96) | 0.023 |
| Hyperkalaemia ≥6.0 | 9,541 | 1,189 | 3.0 | 699 | 42 | 2.3 | 0.00 / 3.14 / 14.49 | 0.90 (0.64-1.26) | 0.527 |
| Hyperkalaemia ≥6.5 | 9,541 | 578 | 1.4 | 699 | 19 | 1.0 | 0.00 / 3.30 / 14.49 | 0.85 (0.52-1.40) | 0.523 |
| Negative control | 11,039 | 1,470 | 3.2 | 1,007 | 46 | 2.0 | 0.00 / 3.08 / 14.49 | 1.00 (0.67-1.48) | 0.986 |
| ^*^Events per 100 person-years | | | | | | | | | |
| ^†^Follow-up in years (minimum / median / maximum) | | | | | | | | | |
