# Supplementary material for: Eplerenone vs. spironolactone in heart failure: long-term cardiorenal and mortality outcomes in a registry-based cohort
Source: Eur Heart J Cardiovasc Pharmacother. 2026 Sep 10;12(7):501–11. doi: 10.1093/ehjcvp/pvag064 (PMC13730579; doi:10.1093/ehjcvp/pvag064)
Supplement: Supplementary file 1 [file pvag064_supplementary_data.zip › TableS3.docx]

| **Exposure** | **Comparison** | **Outcome** | **N at risk** | **Events** | **aHR (95% CI)** | **P** |
| --- | --- | --- | --- | --- | --- | --- |
| Switched MRA (vs. persisted) | Switched | All-cause mortality | 9,967 | 4,898 | 0.80 (0.63–1.00) | 0.048 |
|  | Switched | HF hospitalisation | 7,841 | 2,316 | 1.12 (0.84–1.49) | 0.452 |
|  | Switched | HF hosp + CV death | 7,841 | 3,298 | 1.02 (0.78–1.32) | 0.887 |
|  | Switched | Composite kidney outcome | 7,798 | 1,885 | 1.04 (0.77–1.42) | 0.783 |
|  | Switched | Hyperkalaemia ≥5.5 | 7,968 | 1,470 | 0.64 (0.42–0.95) | 0.028 |
| Discontinued MRA (vs. continued) | Discontinued | All-cause mortality | 9,967 | 4,898 | 1.09 (1.03–1.16) | 0.006 |
|  | Discontinued | HF hospitalisation | 7,841 | 2,316 | 1.12 (1.03–1.23) | 0.012 |
|  | Discontinued | HF hosp + CV death | 7,841 | 3,298 | 1.07 (1.00–1.16) | 0.065 |
|  | Discontinued | Composite kidney outcome | 7,798 | 1,885 | 0.87 (0.78–0.97) | 0.009 |
|  | Discontinued | Hyperkalaemia ≥5.5 | 7,968 | 1,470 | 0.82 (0.73–0.92) | 0.001 |
| Dose change (vs. stable) | Escalation | All-cause mortality | 9,967 | 4,898 | 1.20 (1.03–1.41) | 0.022 |
|  | Reduction | All-cause mortality | 9,967 | 4,898 | 1.31 (1.09–1.57) | 0.004 |
|  | Escalation | HF hospitalisation | 7,841 | 2,316 | 1.02 (0.80–1.30) | 0.879 |
|  | Reduction | HF hospitalisation | 7,841 | 2,316 | 0.83 (0.58–1.17) | 0.285 |
|  | Escalation | HF hosp + CV death | 7,841 | 3,298 | 1.05 (0.86–1.29) | 0.627 |
|  | Reduction | HF hosp + CV death | 7,841 | 3,298 | 0.92 (0.70–1.20) | 0.535 |
|  | Escalation | Composite kidney outcome | 7,798 | 1,885 | 1.14 (0.88–1.47) | 0.321 |
|  | Reduction | Composite kidney outcome | 7,798 | 1,885 | 1.12 (0.79–1.59) | 0.529 |
|  | Escalation | Hyperkalaemia ≥5.5 | 7,968 | 1,470 | 1.24 (0.93–1.63) | 0.138 |
|  | Reduction | Hyperkalaemia ≥5.5 | 7,968 | 1,470 | 0.91 (0.59–1.42) | 0.693 |
