# Supplementary material for: Eplerenone vs. spironolactone in heart failure: long-term cardiorenal and mortality outcomes in a registry-based cohort
Source: Eur Heart J Cardiovasc Pharmacother. 2026 Sep 10;12(7):501–11. doi: 10.1093/ehjcvp/pvag064 (PMC13730579; doi:10.1093/ehjcvp/pvag064)
Supplement: Supplementary file 1 [file pvag064_supplementary_data.zip › TableS4.docx]

| **Dose** | **Spironolactone** | **Eplerenone** |
| --- | --- | --- |
| 25 mg | 9971 (90.3%) | 848 (84.2%) |
| 50 mg | 918 (8.3%) | 138 (13.7%) |
| 100 mg | 59 (0.5%) | 1 (0.1%) |
| Other | 91 (0.8%) | 20 (2.0%) |
